# Supplementary material for: Strain-level genetic diversity of Methylophaga nitratireducenticrescens confers plasticity to denitrification capacity in a methylotrophic marine denitrifying biofilm
Source: PeerJ. 2018 Apr 23;6:e4679. doi: 10.7717/peerj.4679 (PMC5918138; doi:10.7717/peerj.4679)

Figure S1: Schematic of the batch cultures of the denitrifying biofilm.

The original biofilm was dispersed and cultured with Bioflow carriers under anoxic conditions. Every week, only the carriers were transferred in fresh medium and cultured under the same conditions.

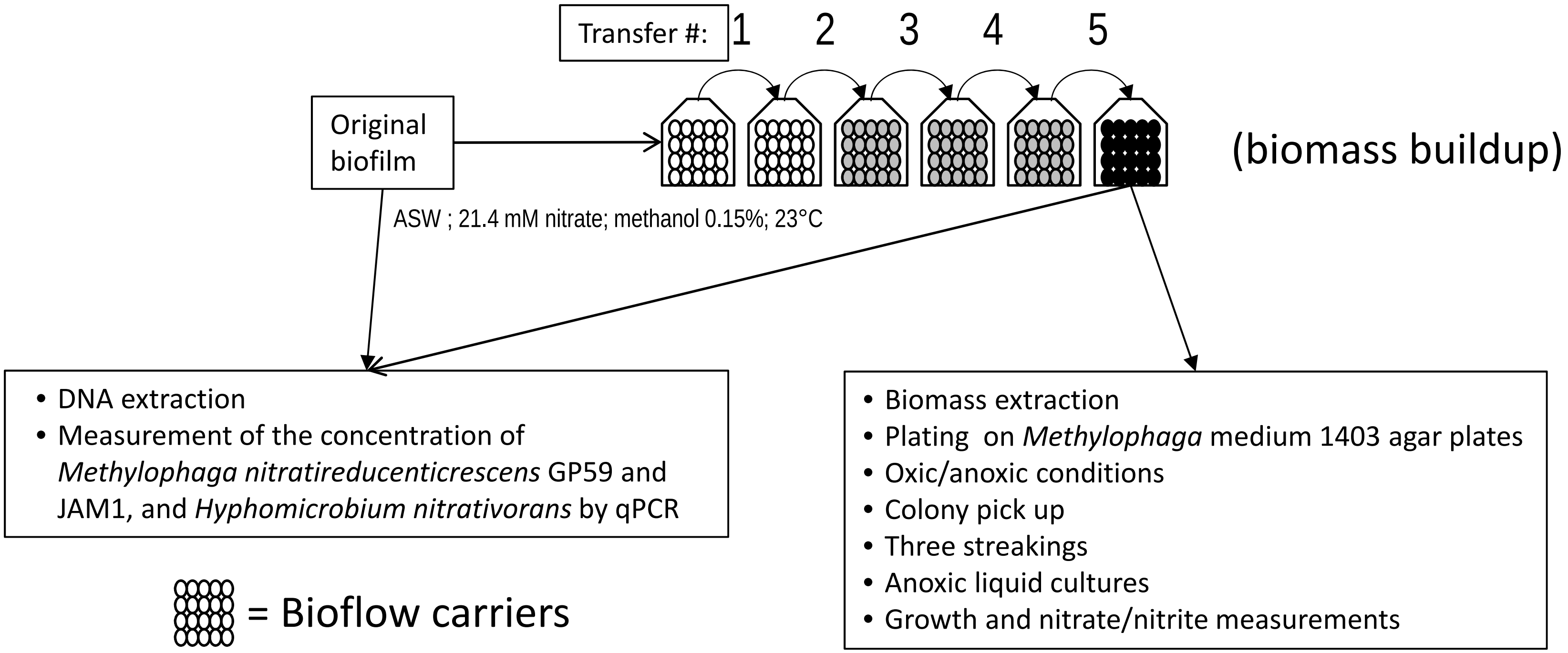

Supplement: Figure S1 [file peerj-06-4679-s003.pdf]
