# Supplementary material for: Strain-level genetic diversity of Methylophaga nitratireducenticrescens confers plasticity to denitrification capacity in a methylotrophic marine denitrifying biofilm
Source: PeerJ. 2018 Apr 23;6:e4679. doi: 10.7717/peerj.4679 (PMC5918138; doi:10.7717/peerj.4679)
Supplement: Figure S2 [file peerj-06-4679-s004.pdf]

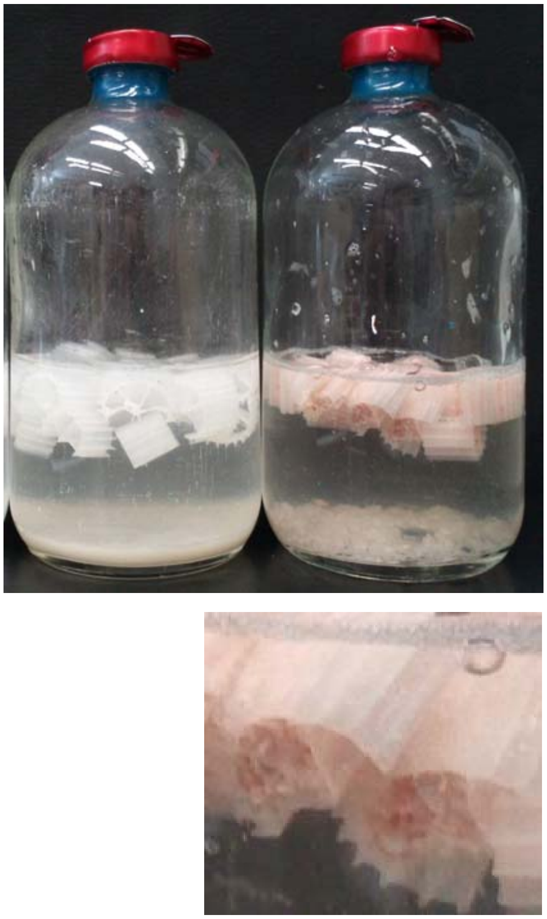

Figure S2: Biofilm batch culture on the Bioflow carriers

-Left vial: The dispersed biofilm before the first transfer.
-Right vial: Biofilm culture after the 5th transfer.
The biofilm is light pink and attached to the carriers.
