# Supplementary material for: Strain-level genetic diversity of Methylophaga nitratireducenticrescens confers plasticity to denitrification capacity in a methylotrophic marine denitrifying biofilm
Source: PeerJ. 2018 Apr 23;6:e4679. doi: 10.7717/peerj.4679 (PMC5918138; doi:10.7717/peerj.4679)
Supplement: Figure S3 [file peerj-06-4679-s005.pdf]

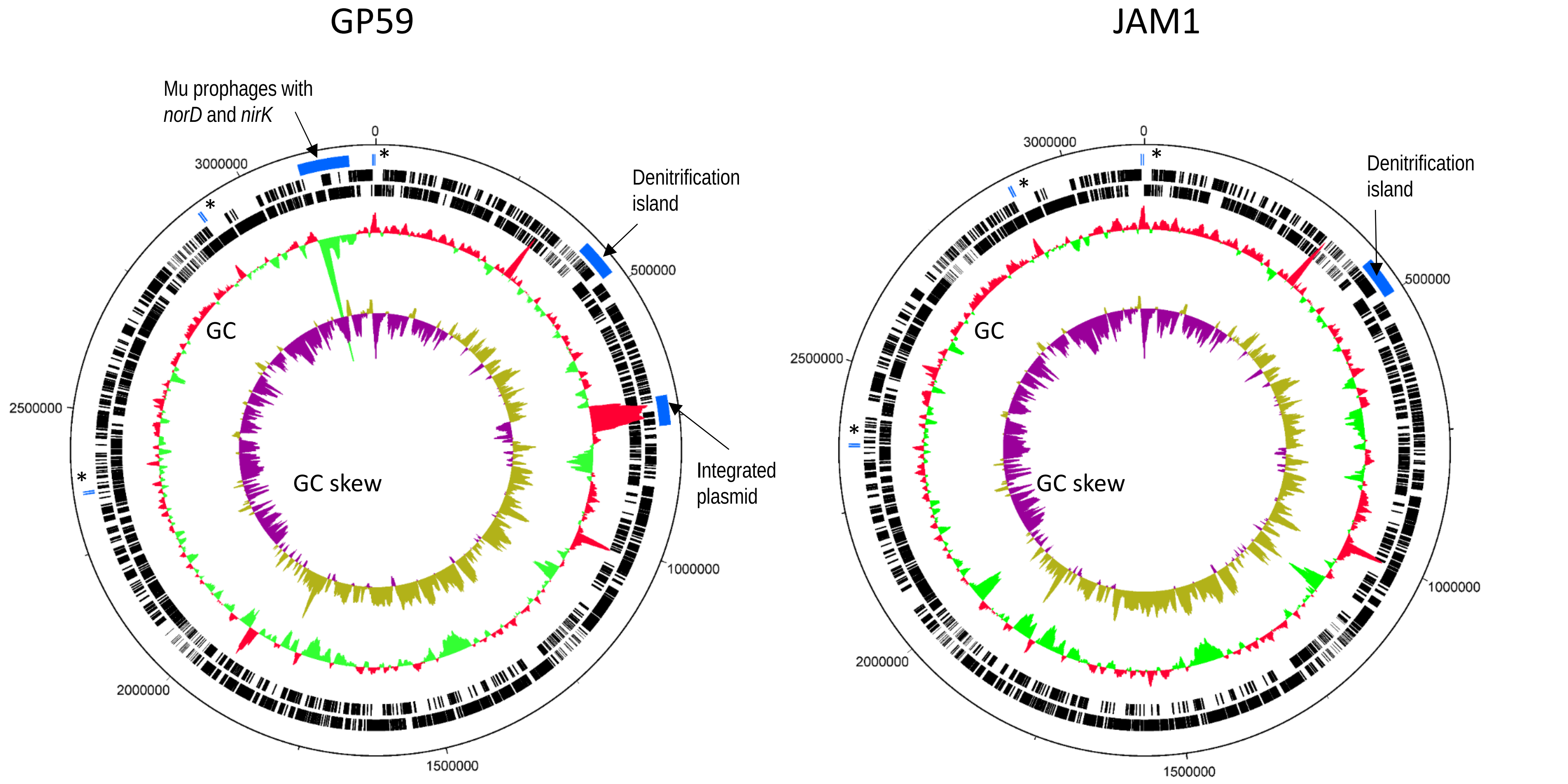

Figure S3: Selected features of the genomes of *Methylophaga nitratireducenticrescens* GP59 (CP021973) and JAM1 (CP003390).

First track (in blue): Specific traits. *: rRNA gene clusters (5S-23S-16S).

Second and third tracks (in black). Coding sequences (plus and minus strands)

Fourth track (red and green): Guanine and Cystosine (GC) content

Fifth track (magenta and olive): GC skew
