# Supplementary material for: Strain-level genetic diversity of Methylophaga nitratireducenticrescens confers plasticity to denitrification capacity in a methylotrophic marine denitrifying biofilm
Source: PeerJ. 2018 Apr 23;6:e4679. doi: 10.7717/peerj.4679 (PMC5918138; doi:10.7717/peerj.4679)

Figure S4: Schematic of selected metabolic pathways in *Methylophaga nitratireducenticrescens* JAM1 and GP59

-Pathways are based on functions deduced by gene annotations (see supplemental Data7)
-The arrow thickness is proportionnal to the level of transcript reads (TPM normalization)
-The red arrows represent GP59 genes with at least 2-fold lower TPM than those of strain JAM1
-The blue arrows represent GP59 genes with at least 2-fold higher TPM than those of strain JAM1

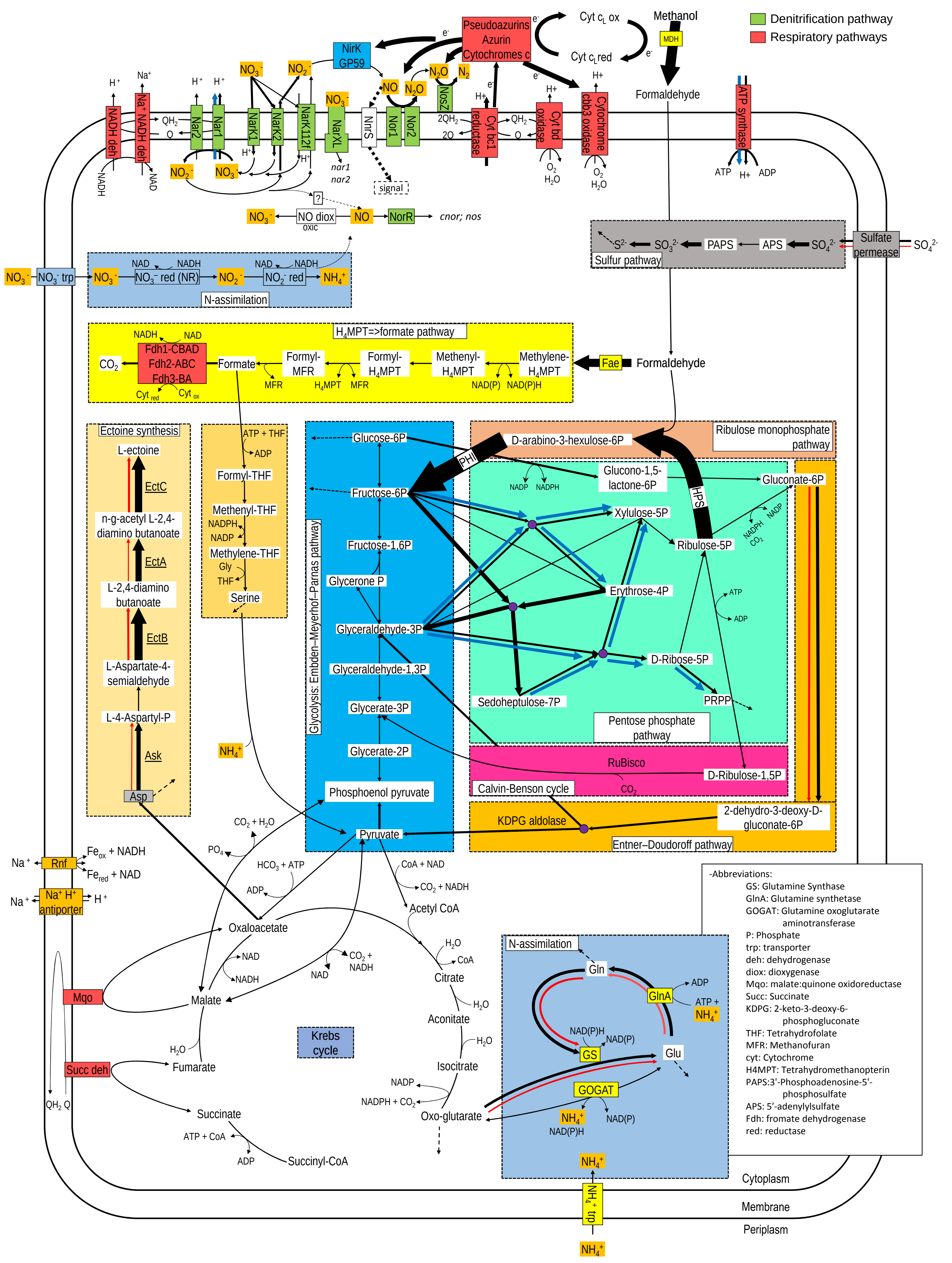

Supplement: Figure S4 [file peerj-06-4679-s006.pdf]
