# Supplementary material for: Strain-level genetic diversity of Methylophaga nitratireducenticrescens confers plasticity to denitrification capacity in a methylotrophic marine denitrifying biofilm
Source: PeerJ. 2018 Apr 23;6:e4679. doi: 10.7717/peerj.4679 (PMC5918138; doi:10.7717/peerj.4679)
Supplement: Data S1 [file peerj-06-4679-s007.docx]

**Supplemental document 1**

**Resequencing the genome of *Methylophaga nitratireducenticrescens* JAM1**

The genome of strain GP59 was sequenced by the PacBio technology that generates long reads allowing an accurate genome assembly. Compared to the JAM1 genome, the GP59 genome had a large inversion of a chromosome region that involved the 5S-23S-16S gene clusters (data not shown). Because the genome of strain JAM1 was sequenced by the Pyrosequencing technology (in 2012), which generates short reads, we revisited the genome assembly of the JAM1 genome provided by the sequencing service. Realignment of the short reads showed ambiguities in the 5S-23S-16S clusters. These clusters are about 5700 nt and almost identical. There were not enough read overlaps to resolve the flanking sequences of two of the 5S-23S-16S clusters in the JAM1 genome. This genome was therefore resequenced by the Pacbio technology, which confirmed the miss-assembly by Pyrosequencing. The combination of Pacbio long reads and Pyrosequencing short reads allowed to resolve several misreads by both technologies to allow correction of several pseudogenes in the JAM1 genome. The new sequence has been updated in GenBank (accession # CP003390.3) and is shorter by 91 nt than the previous version.
