# Supplementary material for: Strain-level genetic diversity of Methylophaga nitratireducenticrescens confers plasticity to denitrification capacity in a methylotrophic marine denitrifying biofilm
Source: PeerJ. 2018 Apr 23;6:e4679. doi: 10.7717/peerj.4679 (PMC5918138; doi:10.7717/peerj.4679)
Supplement: Data S3 [file peerj-06-4679-s009.docx]

**Supplemental document 3**

**Genes encoding copper-containing nitrite reductase in available *Methylophaga* genomes**

During the screening of the protein databases for sequences similar to the GP59-type NirK, other NirK were found in *Methylophaga* genomes with low similarity (30-40%) with the four GP59-type NirK. These NirK are encoded in the genome of *M. frappieri* JAM7 and in 34 genomes of *Methylophaga* sp. retrieved from metagenomics studies (see Table below), all with a deduced amino acid sequence of 436 residues, which is *ca*. 70 residues longer than the GP59-type NirK. These 35 NirK can be grouped in five highly similar sequences (ranging from 87.3-97.9% identity). The extra amino acid residues of these NirKs are explained by the presence at their N-terminal of a transmembrane domain (DUF2070 superfamily). Analyses of protein databases revealed the presence of this type of NirK in other bacteria. Usually, NirK structure contains two cupredoxin domains involved in electron transfer. However, in recent year, two sub-classes NirK have been described with an additional domain, containing an extra cupredoxin domain fused at the N-terminal or a cytochrome *c* at the C-terminal. The exact function of these extra domains remained elusive (1, 2).

1. Eady RR, Antonyuk SV, Hasnain SS. Fresh insight to functioning of selected enzymes of the nitrogen cycle. Curr Opin Chem Biol 2016;31:103-112.

2. Horrell S, Kekilli D, Strange RW, Hough MA. Recent structural insights into the function of copper nitrite reductases. Metallomics 2017;9(11):1470-1482.

Accession number Organism name or Isolate name Location*

1-MAL48203.1 NZMQ01

1-MBP24210.1 PBSF01

1-DEXF01000021.1 UBA3191_contig_7369 172436 to 171078: Frame -1

1-DEMJ01000180.1 UBA2673_contig_173 18053 to 16695: Frame -2

1-DIBK01000048.1 UBA5054_contig_100 2757 to 4115: Frame 3

1-DEMV01000010.1 UBA2661_contig_238 171184 to 169826: Frame -1

1-DELV01000006.1 UBA2687_contig_1555 2758 to 4116: Frame 1

1-DIBB01000005.1 UBA5063_contig_530 2758 to 4116: Frame 1

1-DGNC01000047.1 UBA4502_contig_11048 84825 to 83467: Frame -1

1-DEXY01000015.1 UBA3172 contig_10075 519 to 1877: Frame 3

1-DFMK01000023.1 UBA3595_contig_472 2736 to 4094: Frame 3

1-DFTU01000075.1 UBA4204_contig_13125 6256 to 4898: Frame -1

1-DFMC01000006.1 UBA3603_contig_114 264802 to 263444: Frame -1

1-DCRO01000012.1 UBA1490_contig_391 2758 to 4116: Frame 1

2-DFMM01000015.1 UBA3593_contig_1630 236165 to 237523: Frame 2

2-MAP25618.1 NZSL01

2-DFLS01000006.1 UBA3613_contig_37 121750 to 120392: Frame -1

3-DELM01000286.1 UBA2696_contig_7658 30816 to 32174: Frame 3

3-PHR38619.1 NORP4-NVXT01

3-DELT01000083.1 UBA2689_contig_675 40816 to 42174: Frame 1

4-AFJ01269.1 *Methylophaga frappieri* JAM7

5-DHZD01000003.1 UBA5113_contig_24 22556 to 23899: Frame 2

5-MBN47613.1 PBNB01

5-DEXE01000006.1 UBA3192_contig_48 460606 to 459263: Frame -3

5-MAK65491.1 NZKW01

5-MAY16214.1 PAKV01

5-DCRU01000002.1 UBA1484 contig_23 108078 to 109421: Frame 3

5-DDOK01000005.1 UBA2496contig_79 460204 to 458861: Frame -2

5-DEQJ01000017.1 UBA3369_contig_2015 17759 to 19102: Frame 2

5-DEMX01000017.1 UBA2659_contig_262 130461 to 129118: Frame -2

5-DHZQ01000013.1 UBA5100_contig_10379 189992 to 188649: Frame -1

5-DDOE01000009.1 UBA2502_contig_4 460235 to 458892: Frame -2

5-DIBH01000002.1 UBA5057_contig_181 108018 to 109361: Frame 3

5-DFLN01000001.1 UBA3618_contig_23 108016 to 109359: Frame 1

5-DCSO01000065.1 UBA1464_contig_3717 5807 to 4464: Frame -3

UBA genome sequences can be retrieved from the Bioproject PRJNA348753 at https://www.ncbi.nlm.nih.gov/bioproject/PRJNA348753.

Sequence were grouped by 100% identity. Sequence identity between each group ranges from 87.3 to 97.9%.

*NirK sequences were retrieved from GenBank annotation at NCBI except for UBA genome sequences where the contig sequences were six-frame translated (>200 amino acid, no start codon specified) and BlastP with the GP59 NirK.
