# Supplementary material for: Strain-level genetic diversity of Methylophaga nitratireducenticrescens confers plasticity to denitrification capacity in a methylotrophic marine denitrifying biofilm
Source: PeerJ. 2018 Apr 23;6:e4679. doi: 10.7717/peerj.4679 (PMC5918138; doi:10.7717/peerj.4679)
Supplement: Data S5 [file peerj-06-4679-s011.pdf]

Supplemental document 5: Plasmid maps

- Extraction of plasmid DNA from strain GP59

- *Methylophaga nitratireducenticrescens* GP59
  - Plasmid pGP32 (GenBank accession # CP021975)
  - Plasmid pGP34 (CP021974)

- *Methylophaga frappieri* JAM7
  - Plasmid pJAM7-48 (CP003381)

Extraction of plasmid DNA from strain GP59

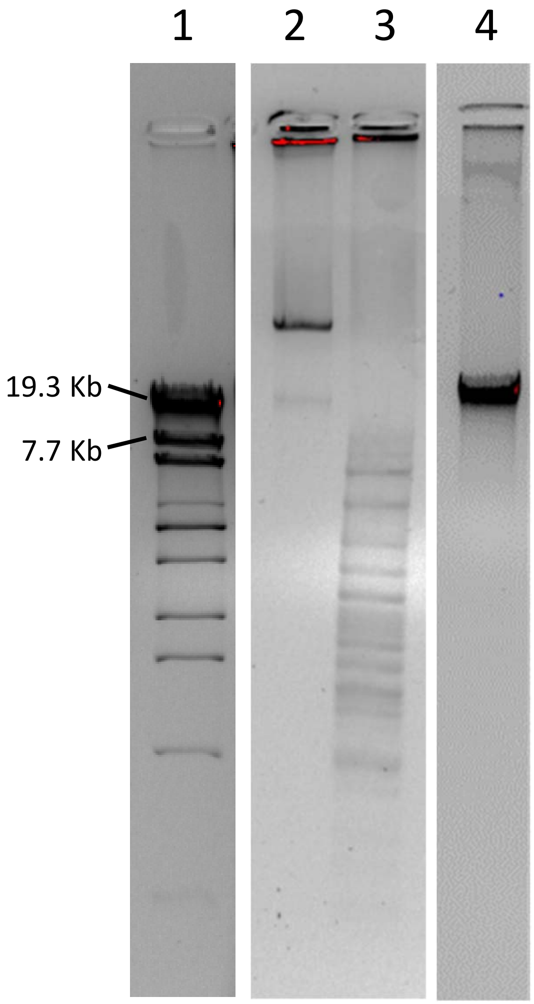

1- Marker
2- Undigested plasmid DNA
3- Plasmid DNA digested with *Rsa*I
4- Genomic DNA

Plasmid DNA was extracted from GP59 cultures by the alkaline lysis protocol, digested with the restriction endonuclease *Rsa*I and ran on a 1% agarose gel electrophoresis. The gel was stained with GelRed to reveal DNA (1).

1-Sambrook JF, Russell DW (ed.). 2001. Molecular Cloning: A Laboratory Manual. Cold Spring Harbor Laboratory Press

Gene annotations were performed by RAST. Further analyses were performed by BLASTP in protein data bases.
Type IV secretion system nomenclature (Vir) is based on the T-DNA transfer system as described by Christie PJ (Mol Microbio 40-2 294-305 2001)

- CsrA: Carbon storage regulator
- PilL: conjugative pilus protein
- ParAB: plasmid partitioning protein
- RepB: plasmid replication initiator protein
- XerD: Site-specific recombinase
- AraC: transcriptional regulator
- RadC: DNA repair protein
- MarR: transcriptional regulator
- FtsH: cell division protein, ATP-dependent zinc metalloprotease

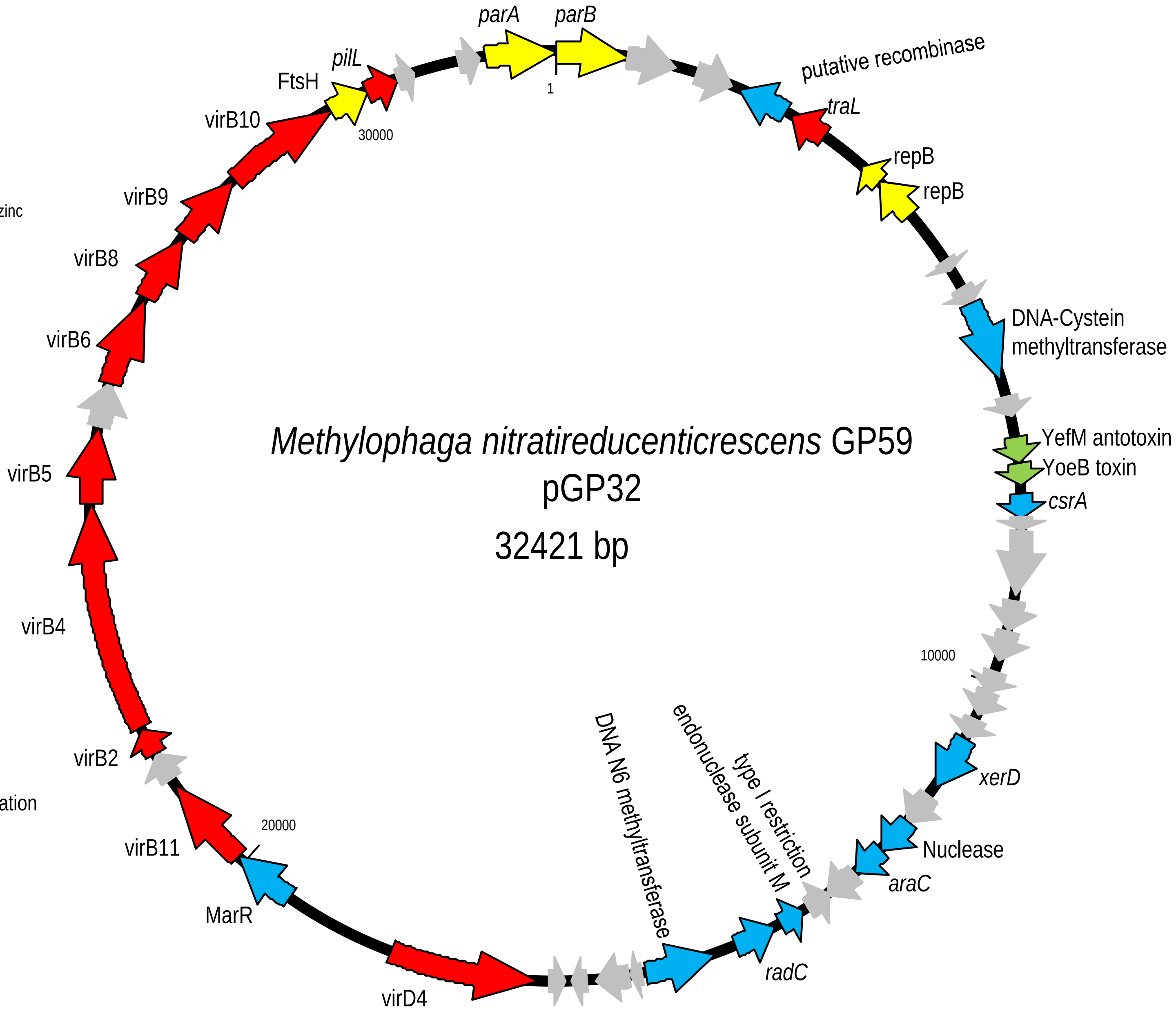

- Stability, plasmid partition, replication
- Type IV secretion system
- Others
- toxin/Antitoxin
- Hypothetical protein

Gene annotations were performed by RAST. Further analyses were performed by BLASTP, in protein data bases
Type IV secretion system nomenclature (Vir) is based on the T-DNA transfer system as described by Christie PJ (Mol Microbio 40-2 294-305 2001)

- CsrA: Carbon storage regulator
- SinI: Restriction endonuclease
- FlgH: Flagellar basal body L-ring protein

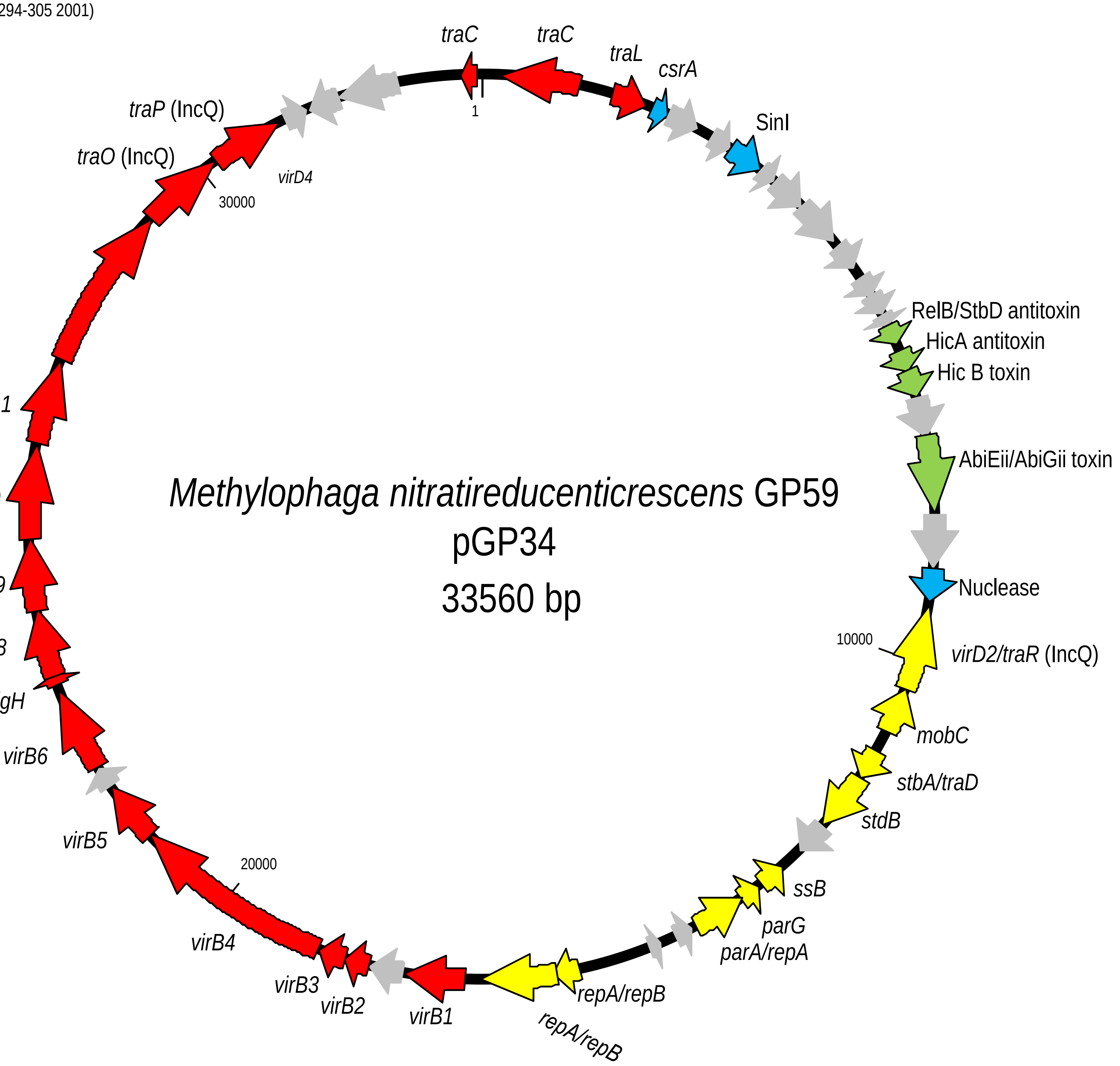

- Stability, plasmid partition, replication
- Type IV secretion system
- Others
- Toxin/Antitoxin
- Hypothetical protein

Gene annotations were performed by GenBank (NCBI) and by RAST. Further analyses were performed by BLASTP in protein databases.
Gene nomenclature is based on the *Pseudomonas aeruginosa* IncP alpha plasmid pBS228 (Haines,A.S., Jones,K., Batt,S.M., Kosheleva,I.A. and Thomas,C.M. Sequence of plasmid pBS228 and reconstruction of the Incp-1alpha phylogeny plasmid 58 (1), 76-83 (2007), except for the « Others » which were deduced from protein database analysis (BLASTP).

- CysZ: putative sulfate transporter
- Trc Reg: Transcriptional regulator
- Tnp: Transposase
- DnaQ-like: DNA polymerase III, epsilon subunit-related 3'-5' exonuclease

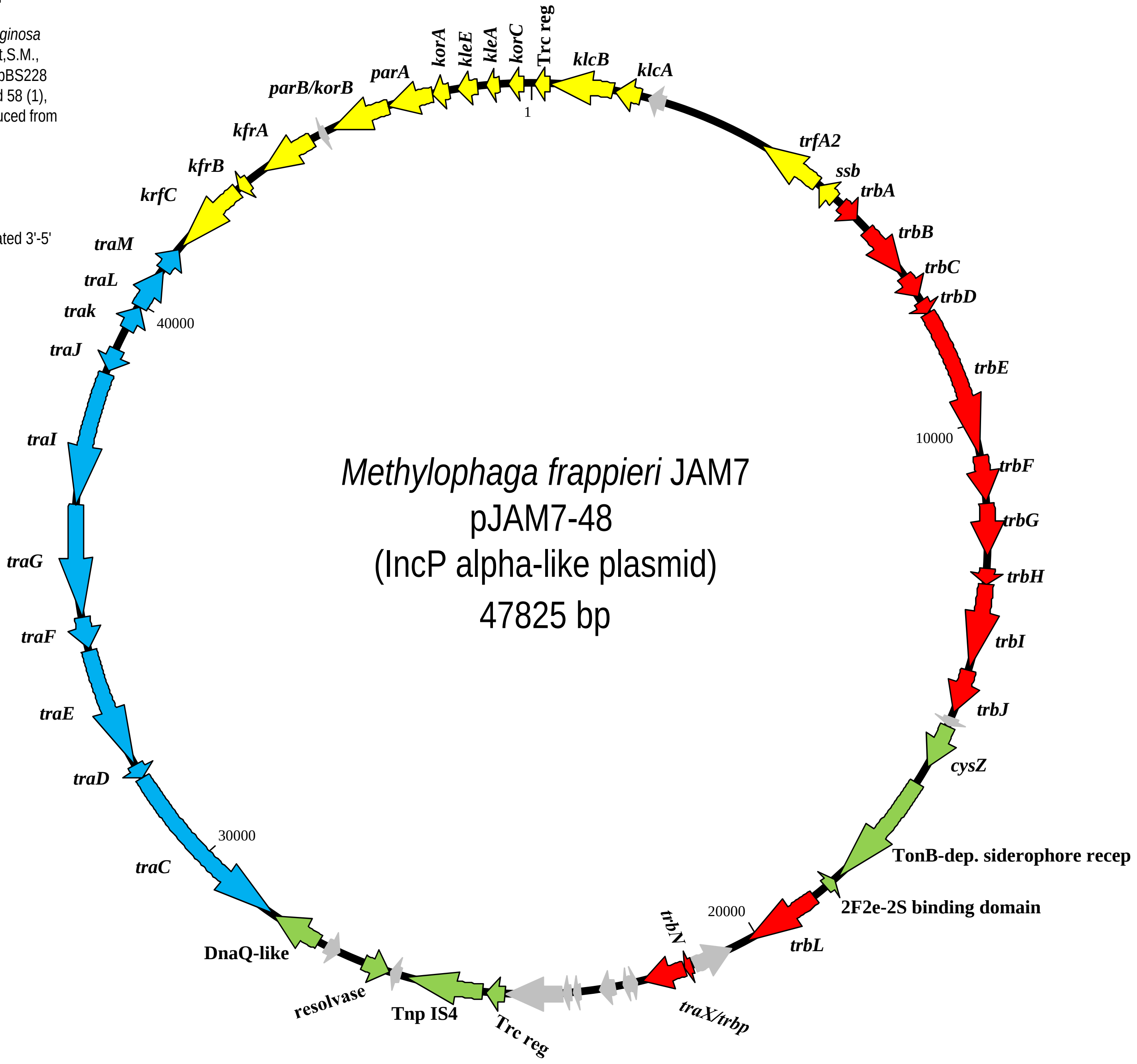

- Stability, plasmid partition, replication
- Tra1: transfer system
- Tra2: transfer system
- Others
- Hypothetical protein
